# Impaired Innate Immunity in *Tlr4* ^−/−^ Mice but Preserved CD8^+^ T Cell Responses against *Trypanosoma cruzi* in *Tlr4*-, *Tlr2*-, *Tlr9*- or *Myd88*-Deficient Mice

**DOI:** 10.1371/journal.ppat.1000870

**Published:** 2010-04-29

**Authors:** Ana-Carolina Oliveira, Bruna C. de Alencar, Fanny Tzelepis, Weberton Klezewsky, Raquel N. da Silva, Fabieni S. Neves, Gisele S. Cavalcanti, Silvia Boscardin, Marise P. Nunes, Marcelo F. Santiago, Alberto Nóbrega, Maurício M. Rodrigues, Maria Bellio

**Affiliations:** 1 Instituto de Microbiologia Prof. Paulo de Góes, Universidade Federal do Rio de Janeiro (UFRJ), Rio de Janeiro, Rio de Janeiro, Brazil; 2 Centro Interdisciplinar de Terapia Gênica (CINTERGEN), Universidade Federal de São Paulo (UNIFESP), São Paulo, São Paulo, Brazil; 3 Departamento de Parasitologia, Instituto de Ciências Biomédicas, Universidade de São Paulo, São Paulo, São Paulo, Brazil; 4 Instituto Osvaldo Cruz (IOC/FIOCRUZ) Rio de Janeiro, Rio de Janeiro, Brazil; 5 Instituto de Biofísica Carlos Chagas Filho (UFRJ), Rio de Janeiro, Rio de Janeiro, Brazil; Imperial College London, United Kingdom

## Abstract

The murine model of *T. cruzi* infection has provided compelling evidence that development of host resistance against intracellular protozoans critically depends on the activation of members of the Toll-like receptor (TLR) family via the MyD88 adaptor molecule. However, the possibility that TLR/MyD88 signaling pathways also control the induction of immunoprotective CD8^+^ T cell-mediated effector functions has not been investigated to date. We addressed this question by measuring the frequencies of IFN-γ secreting CD8^+^ T cells specific for H-2K^b^-restricted immunodominant peptides as well as the *in vivo* Ag-specific cytotoxic response in infected animals that are deficient either in TLR2, TLR4, TLR9 or MyD88 signaling pathways. Strikingly, we found that *T. cruzi*-infected *Tlr2^−/−^*, *Tlr4^−/−^*, *Tlr9^−/^* ^−^ or *Myd88^−/−^* mice generated both specific cytotoxic responses and IFN-γ secreting CD8^+^ T cells at levels comparable to WT mice, although the frequency of IFN-γ^+^CD4^+^ cells was diminished in infected *Myd88^−/−^* mice. We also analyzed the efficiency of TLR4-driven immune responses against *T. cruzi* using TLR4-deficient mice on the C57BL genetic background (B6 and B10). Our studies demonstrated that TLR4 signaling is required for optimal production of IFN-γ, TNF-α and nitric oxide (NO) in the spleen of infected animals and, as a consequence, *Tlr4^−/−^* mice display higher parasitemia levels. Collectively, our results indicate that TLR4, as well as previously shown for TLR2, TLR9 and MyD88, contributes to the innate immune response and, consequently, resistance in the acute phase of infection, although each of these pathways is not individually essential for the generation of class I-restricted responses against *T. cruzi*.

## Author Summary

Innate and acquired immune responses are triggered during infection with *T. cruzi*, the etiologic agent of Chagas' disease, and are critical for host survival. Parasite burden is usually controlled by the time the adaptive response becomes operational. Nevertheless, *T. cruzi* manages to subsist within intracellular niches and establishes a chronic infection, leading to the development of cardiomyopathy in approximately one-third of infected individuals. Recently, Toll-like receptors (TLRs) have been shown to recognize *T. cruzi* molecules and mice lacking MyD88, the key adaptor for most TLRs, are extremely susceptible to infection. Although TLRs are known to link innate and adaptive responses, their role in the establishment of crucial effector mechanisms mediated by CD8^+^ T cells during *T. cruzi* infection has not yet been determined. We analyzed the induction of IFN-γ and cytotoxic activity *in vivo* in TLR2-, TLR4-, TLR9- or MyD88-deficient mice during infection, and found intact responses compared to WT mice. We also demonstrated that TLR4 is required for optimal production of inflammatory cytokines and nitric oxide and, consequently, for a better control of parasitemia levels. Understanding how TLR activation leads to resistance to infection might contribute to the development of better strategies to improve immune responses against this pathogen.

## Introduction

*T. cruzi* is an intracellular protozoan parasite that causes Chagas' disease, an endemic disorder affecting 16–20 million people which remains a health problem in Latin America. Although both innate and acquired immune responses are triggered during early infection and are critical for host survival, around 5% of individuals die due to myocarditis during the acute phase of the disease. In most cases, despite of the immune response, *T. cruzi* manages to subsist within the host and in approximately 30% of infected individuals it establishes a lifelong chronic illness presenting different clinical forms, including cardiomyopathy and megasyndrome in the gut [1]. Immunopathology due to parasite persistence is considered a key element in the development of chagasic cardiomyopathy, although a secondary role for autoimmunity is not completely excluded.

Different members of the family of Toll-like receptors (TLRs), by recognizing diverse pathogen-associated molecular patterns (PAMPs) of bacterial, viral, fungal, and protozoan origin trigger the activation of innate immunity and the subsequent development of Ag-specific adaptive immunity [2]. To date, TLR2, TLR4, and TLR9 have been implicated in recognition of different *T. cruzi*-derived PAMPs [3]–[6]. TLR2 recognizes GPI-anchors of mucin-like proteins and the *T. cruzi*-released protein Tc52 [3], [4], whereas TLR4 is responsible for recognition of free glycoinositolphospholipids [5] and TLR9 is involved in recognition of the CpG motif present in *T. cruzi* DNA [6]. Mice deficient in MyD88, the adaptor molecule required for signaling events by most TLRs as well as IL-1R and IL-18R, show greatly enhanced susceptibility to infection with this protozoan parasite [7]. The susceptibility to infection of *Tlr2^−/^*, *Tlr9^−/−^* and *Tlr2^−/^Tlr9^−/−^* double knockout mice (all in the C57BL/6 background) has also been analyzed [6], [7]. Interestingly, although mice simultaneously lacking TLR2 and TLR9 are highly vulnerable to infection, their mortality rate is still less than that of *Myd88^−/−^* mice, pointing to the involvement of other TLRs and/or IL-1/IL-18 in the control of mortality.

In addition to MyD88-dependent activation, another transduction pathway is involved in signaling through TLR3 and TLR4. This pathway is mediated by the TIR domain-containing adaptor inducing IFN-γ (TRIF). Interestingly, *Myd88^−/−^Trif^−/−^* and *Myd88^−/−^Ifnar^−/−^* double knock out mice were even more sensitive to *in vivo* infection with *T. cruzi* than *Myd88^−/−^* mice, indicating that in addition to MyD88-dependent induction of proinflammatory cytokines, the TRIF-dependent production of type I IFN also contributes resistance to *T. cruzi* infection [8]. In accord with this observation, we have previously demonstrated that the lack of expression of functional TLR4 in mice of C3H background caused higher parasitemia and accelerated mortality to *T. cruzi* infection [5], although the mechanisms by which this occurs are not yet fully determined. However, since C3H WT mice are known to be more susceptible to *T. cruzi* infection when compared to mice of the C57BL strains, the direct comparison between the levels of susceptibility of C3H/HeJ (TLR4-deficient) mice and the other above mentioned *Tlr^−/−^* and *Myd88^−/−^* mice is difficult to interpret.

Therefore, one of the aims of the present work was to analyze the role of TLR4 in the C57BL background in the innate response to *T. cruzi*. For this, host cell invasion, parasite survival and release from infected macrophages, as well as nitric oxide (NO) production were quantified in C57BL/6 (WT) and TLR4-deficient cell cultures. We also evaluated the contribution of TLR4 to the *in vivo* control of parasitemia levels and survival, as well as to IFN-γ and TNF-α production in the B6 and B10 backgrounds. Importantly, the participation of TLR2, TLR4, TLR9 and MyD88 in the induction of crucial effector mechanisms of the adaptive response against *T. cruzi* was also investigated, measured as the Ag-specific IFN-γ production and cytotoxic response mediated by CD8^+^ T cells in infected mice.

## Results

### Defective early trypanosomicidal mechanism in TLR4-deficient macrophages

**Figure 1:**
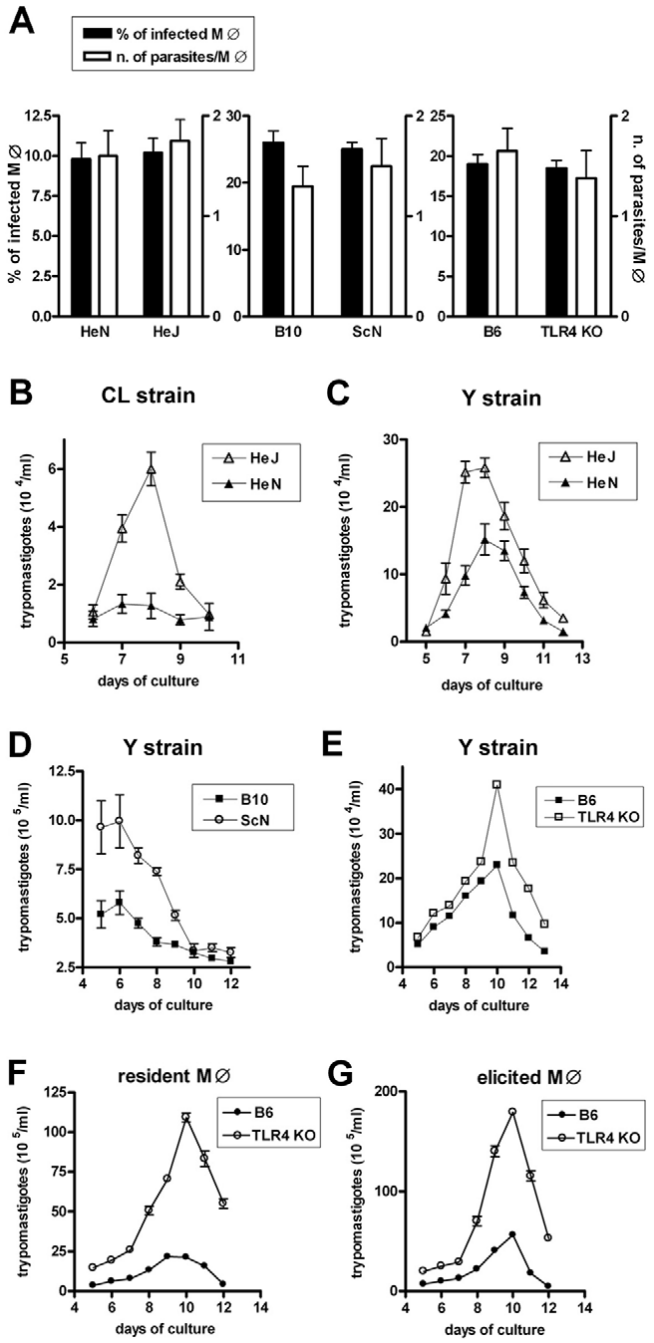
Equal invasion rate but increased growth of *T. cruzi* in TLR4-deficient macrophages. (**A**) 10^5^ resident peritoneal macrophages from TLR4-deficient strains C3H/HeJ, C57BL/10ScN, or TLR4^−/−^ mice and from their respective WT controls (C3H/HePas, C57BL/10, or C57BL/6) were cultured in the presence of blood form trypomastigotes of the Y strain in a 1∶10 (macrophage:trypomastigotes) ratio, for 1 h, as described in Materials and Methods. After that period, extracellular trypomastigotes were removed by washing and the cells were fixed and stained with Giemsa. The percentage of infected macrophages and the intracellular parasite numbers in 100 macrophages were counted under a light microscope. (**B–G**) Cells were infected with blood form trypomastigotes of the Y (**C–G**) or CL strains (**B**) as described in (**A**). Thioglycollate elicited peritoneal macrophages from B6 and TLR4^−/−^ mice were cultured in (**G**). After removal of extracellular trypomastigotes by extensive washing, cultures were continued for several days and the number of trypomastigotes released into the supernatants was determined daily from day 5 on. Each data point is expressed as the mean ± SEM of triplicates and experiments shown are representative of at least two independent experiments.

In order to compare the anti-*T. cruzi* microbicidal activity of WT and TLR4-deficient macrophages, it was first necessary to investigate whether the infection rate and parasite load were equivalent in both cases. Therefore we first compared the capacity of *T. cruzi* trypomastigotes to infect TLR4-deficient and WT macrophage (MO) cultures in three different genetic backgrounds: C3H, C57BL/10 and C57BL/6. Strains C3H/HeJ and C57BL/10ScN are natural mutants in which the *Tlr4* gene suffered mutations that result either in a residue substitution (P712H), rendering the receptor non-functional, or a deletion, with non-expression of TLR4, respectively [9]. Engineered *Tlr4* ^−/−^ in the B6 background was also previously described [10]. As shown in Figure 1A, no difference in the percentage of infected macrophages or in the number of parasites per macrophage after one hour of infection could be detected between cultures from the TLR4-deficient strains and their respective WT controls. However, when non-internalized parasites were extensively washed out after 1 h of interaction and the cultures were left to continue for three more hours, a significantly higher percentage of infected MO was found in TLR4-mutant cultures (Fig. S1). This result suggests the existence of an early microbicidal mechanism which is dependent on a functional TLR4. In agreement with that, the number of trypomastigotes released in the supernatant after the parasite completes its intracellular cycle in long-term cultures is significantly higher in the TLR4-deficient MO cultures (Fig. 1B–G). This is true for *T. cruzi* Y (Fig. 1C–G) and CL strains (Fig. 1B) and for both resident and elicited macrophages (Fig. 1F and 1G). Together these results indicated that although cell invasion by the parasite is not affected by the absence of a functional TLR4, *T. cruzi* growth is favored in TLR4-deficient MO, possibly due to a defective early anti-trypanosomacidal mechanism in TLR4-deficient MO.

### TLR4 and *T. cruzi* colocalization in lysosomes of host cells

**Figure 2:**
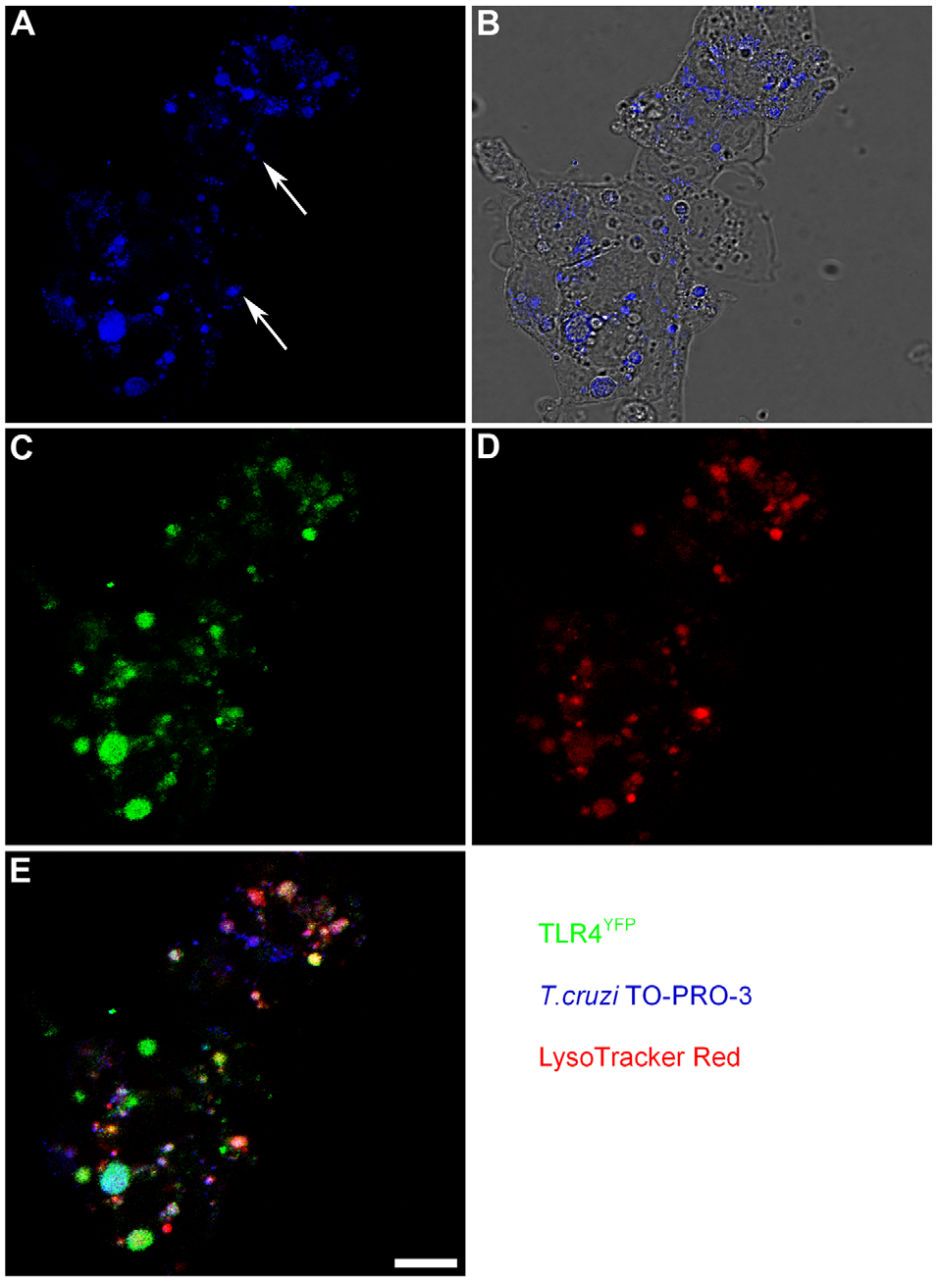
TLR4 is associated with *T. cruzi* that entered lysosomal compartments. HEK293-TLR4^YFP^ cells were infected with TO-PRO-3 labeled trypomastigotes at 1∶10 ratio. 2 h later, non-internalized parasites were removed by washing and cells were stained with LysoTracker Red for 0.5 h. Confocal sections were collected and the intracellular distribution of TLR4^YFP^ and *T. cruzi* was analyzed. (**A**) TO-PRO-3-labeled trypomastigotes (blue) are uniformly dispersed inside the infected cells as shown by phase contrast microscopy (**B**). In (**C**) and (**D**) we see the subcellular distribution of TLR4^YFP^ (green) and LysoTracker-stained lysosomes (red) in HEK293-infected cells, respectively. Note in (**E**) several trypomastigotes (blue) co-localized with TLR4 (green) inside lysosomes (red). White arrows indicate two trypomastigotes, for illustration. Calibration bar: 20 µm.

The expression of fluorescent TLR4 in cell lines allowed us to map TLR4 subcellular location, demonstrating its presence on the cellular surface and in the Golgi, similar to the TLR4 distribution observed in human monocytes [11]. It is also known that early after cell invasion the *T. cruzi* localizes in a host cell vacuole which fuses with peripheral lysosomes. HEK293 cells stably transfected with TLR4-yellow fluorescent protein and MD-2 (HEK-TLR4^YFP^) were infected with labeled *T. cruzi* trypomastigotes and 2.5 h later we analyzed parasite-TLR4 co-localization by confocal microscopy. Staining these infected cells with a lysosome probe also revealed that *T. cruzi*-TLR4 co-localization occurs in acidic compartments (Figure 2).

### iNOS inhibition in infected WT macrophages leads to an increase in the number of released trypomastigotes equaling non-treated *Tlr4^−/−^* macrophages

**Figure 3:**
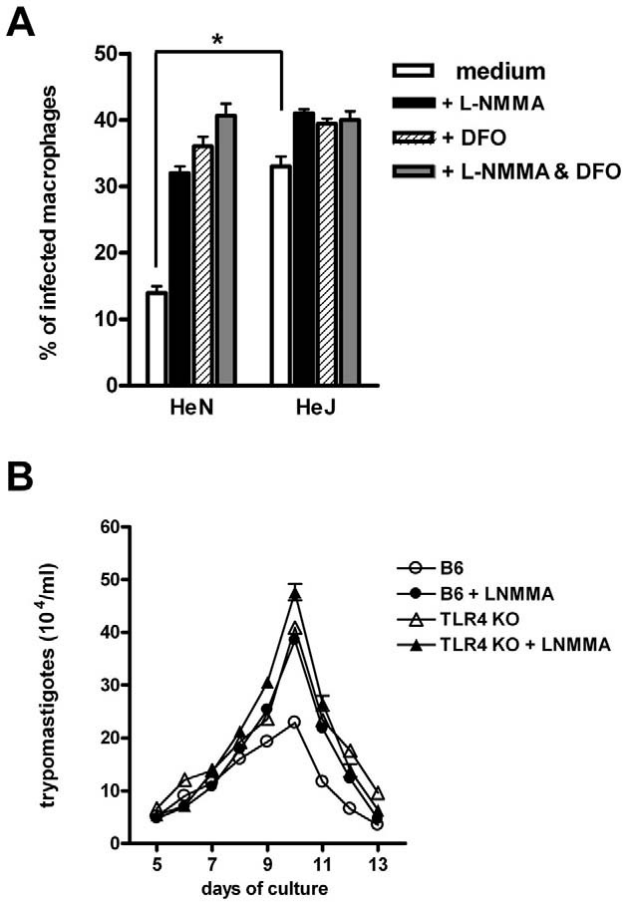
Defective early trypanosomacidal mechanism in TLR4-deficient macrophages. (**A**) Resident macrophages from C3H/HePas (wt) and C3H/HeJ (*Tlr4^d/d^*) mice were infected with blood form trypomastigotes of the Y strain in the presence of L-NMMA (1 mM) and/or DFO (100 µM), according to the legend and as detailed in the Materials and Methods section. After 4 h of culture, cells were fixed and stained with Giemsa. Asterisk (*) indicates that the percentage of infected MO is significantly different (p<0.05) between WT and TLR4-deficient cultures without treatment. (**B**) Resident peritoneal macrophages from B6 and *Tlr4^−/−^* mice were infected with blood form trypomastigotes of the Y strain, as described in Fig. 1, with (closed symbols) or without (open symbols) L-NMMA (1 mM). The number of trypomastigotes released into the supernatants was assessed daily from day 5 on. Each data point is expressed as the mean ± SEM of triplicates and experiments shown are representative of at least two independent ones.

Both NO and reactive oxygen species (ROS) have been shown to mediate *T. cruzi* killing [12]–[15]. Thus, we next analyzed the effects of adding NO and/or ROS inhibitors to the MO cultures during infection. Figure 3A shows that the addition of desferroxamine (DFO), an iron chelator which can also act as a free radical scavenger [16], causes a significant increase in the percentage of infected WT MO. This treatment abolishes the otherwise significant difference found in the percentage of infected MO between the non-treated WT and TLR4-deficient cultures. The same results are obtained when the inducible NO synthase (iNOS) inhibitor L-NMMA, or the combination of DFO and L-NMMA are added to these cultures. In order to further confirm the relevance of this early microbicidal mechanism, absent in TLR4-deficient MO, we tested the effect of inhibiting NO production in long term MO cultures, in which the number of parasites released by infected cells in the supernatant was evaluated several days after initial infection. As shown in Figure 3B, while iNOS inhibition had no effect in the number of parasites released by *Tlr4^−/−^* MO cultures, the addition of L-NMMA to the infected WT MO raised the number of trypomastigotes found in the supernatants to the levels observed in the *Tlr4^−/−^* MO cultures. In contrast, the addition of rIFN-γ an iNOS inducer to the MO cultures from the beginning of infection reduces the quantity of free trypomastigotes and results in equal numbers of released parasites from WT or TLR4-deficient MO (Fig. S2). Together, these results strongly suggest that the early trypanosomacidal mechanism absent in TLR4-deficient MO depends on ROS and NO induction.

### Higher parasitemia and mortality in TLR4-deficient mice of C57BL background

**Figure 4:**
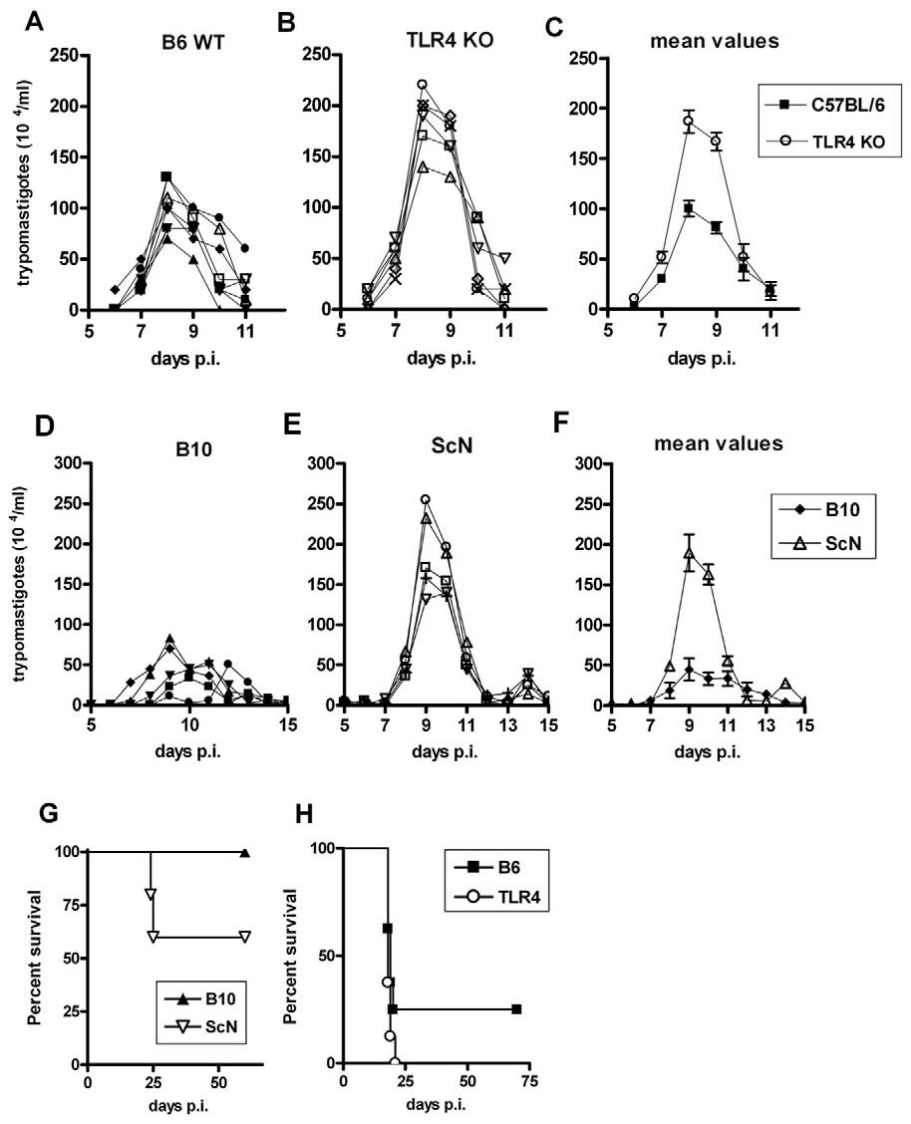
Enhanced susceptibility of *Tlr4^−/−^* mice to infection with *T. cruzi* parasites. Groups of 5 or more male 6*-*wk*-old* mice from B6 (wt) (**A, C and H**), *Tlr4^−/−^* (**B, C and H**), B10 (wt) (**D, F and G**) and C57BL/10ScN (**E, F and G**) strains of mice were infected with 2×10^3^ trypomastigote of the Y strain of *T. cruzi* and parasitemia (**A–F**) as well as mortality (**G and H**) were assessed daily. Parasitemia values of each mouse are shown in (**A, B, D and E**). (**C**) and (**F**) represent the mean values of individual parasitemia shown in (**A and B**), and (**D and E**), respectively. Mortality curves (**G**) were statistically different (p<0.05). The results shown are representative of three independent experiments.

We next compared parasitemia and mortality between different pairs of WT and TLR4-deficient mice (B10 or B6 versus B10/ScN or *Tlr4* KO, respectively), after i.p. infection with 2×10^3^ *T. cruzi* strain Y bloodstream trypomastigotes. Results in Figures 4A-C and 4D-F show that in both cases we found significantly higher parasitemia levels in TLR4-deficient mice, although the levels of parasites in the blood returned to very low or undetectable levels by day 11–12 post-infection and did not rise again, differently from what was previously described for C3H/HeJ mice, in which parasitemia levels were not controlled after day 15 pi [5]. We further monitored the mortality after infection and found that TLR4-deficiency in both B10 and B6 backgrounds results in higher lethality. Statistically significant differences, however, were consistently found only when comparing B10 and B10/ScN mice, while results with B6 and *Tlr4^−^* ^/−^ were more variable and did not reach statistical significance (Figure 4G and H). Of note, these results were obtained in male mice of 6–7 weeks of age, while in older TLR4-deficient mice the higher susceptibility could not be observed (data not shown). We have also performed experiments with lower (10^2^) and higher (10^4^ and 10^5^) doses of infective *T. cruzi* forms/mice obtaining the same results (data not shown). Therefore, mice lacking TLR4 expression in a C57BL genetic background are more sensitive than their WT controls to infection with *T. cruzi*, although these strains do not display the uncontrolled parasitemia and the remarkable earlier mortality previously observed in the TLR4-mutant C3H/HeJ mice [5].

### Splenocytes from infected TLR4-deficient mice produce lower NO levels than WT mice. NO inhibition in vivo makes WT and TLR4-deficient mice equally susceptible to infection

**Figure 5:**
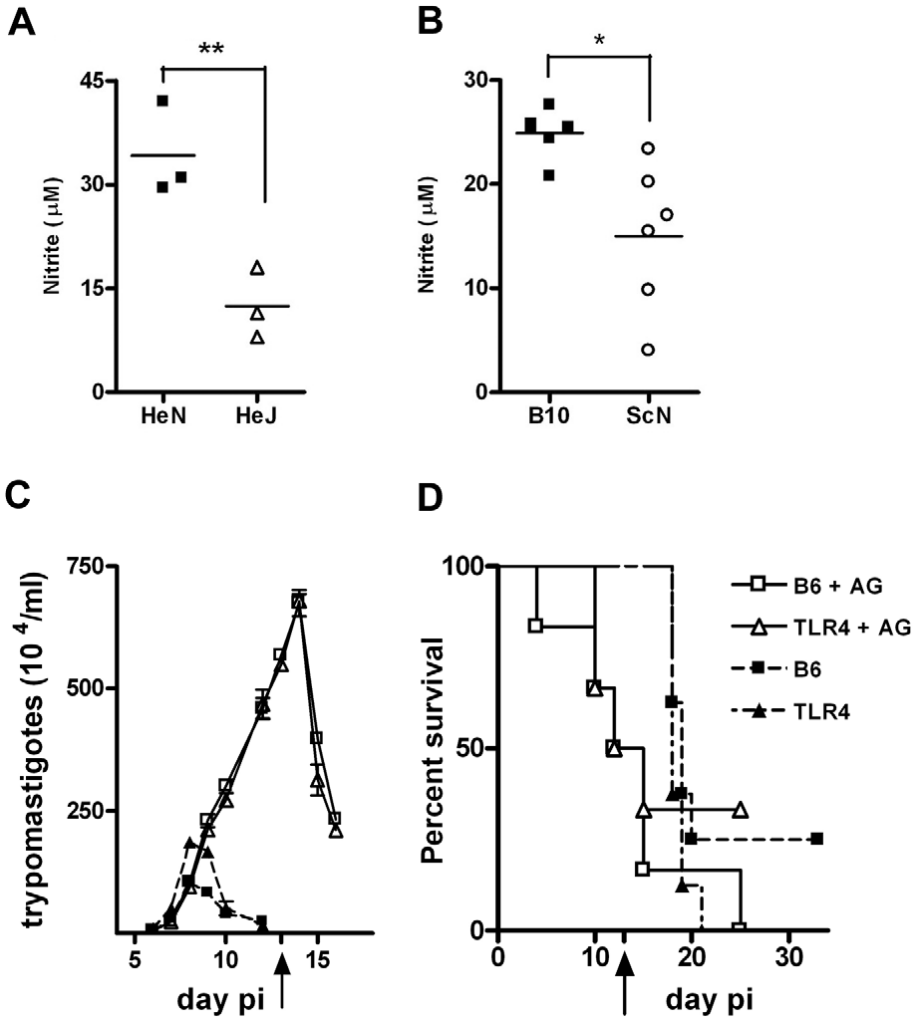
*In vivo* blockage of NO production turns WT and *Tlr4^−/−^* mice equally susceptible to infection with *T. cruzi*. (**A**) and (**B**) show the levels of nitrite in the supernatant from splenocyte cultures from TLR4-deficient C3H/HeJ and C57BL/10ScN, respectively, at 10 days post infection (pi) with *T. cruzi* and compared to their WT controls. Asterisk (*) indicates that difference is statistically significant (p≤0.01). (**C**) Parasitemia and (**D**) Survival curves of infected B6 and *Tlr4^−/−^* mice treated (or not) every other day with aminoguanidine (AG) from the beginning of infection until day 13 pi. Black arrows in (C) and (D) indicate the day when the treatment was stopped. The results shown are representative of two different experiments.

We then analyzed whether a lower NO production by the infected TLR4-deficient mice could explain their higher susceptibility to infection as suggested by the results obtained *in vitro*. In accordance with that hypothesis, the production of NO (inferred from nitrite levels in the supernatants) by spleen cells from infected TLR4-deficient mice was significantly reduced when compared with NO released by spleen cells from infected WT mice at day 10 post-infection (Figure 5A and B). Nitrite levels are also lower in the sera of TLR4-deficient mice, compared to WT animals, at this time point of infection (data not shown). Furthermore, the *in vivo* blockade of NO production in *T. cruzi* infected animals, by injection of the inducible NO synthase (iNOS) inhibitor aminoguanidine (AG) in the early phase of acute infection, brought the parasitemia and mortality of treated WT mice to the same levels obtained in treated *Tlr4* ^−/−^ animals (Figure 5C and D). In animals injected every other day with AG, following a previously reported protocol [17], parasitemia kept rising until treatment was stopped on day 13 pi, attaining 3 and 7 fold higher levels of what was usually observed in *Tlr4* ^−/−^ and WT non-treated animals, respectively (Figure 5C). This is due to the prevention of all NO generation, as for example in response to TLR2 and/or TLR9 signaling pathways, rather than exclusive inhibition of NO triggered by TLR4 engagement. Also, while non-treated infected animals usually die only after day 20 pi, earlier mortality was observed among AG-treated mice, with 50% mortality in both *Tlr4* ^−/−^ and WT AG-treated groups by day 12 pi (Figure 5D). Hence, these results suggest that the lower NO production due to the absence of TLR4 expression during the early phase of infection with *T. cruzi* is responsible for the higher sensitivity observed in *Tlr4* ^−/−^ mice.

### The frequency of IFN-γ producing CD8^+^ T cells is not reduced in infected TLR4-deficient mice, although total spleen levels of IFN-γ and TNF-α are

**Figure 6:**
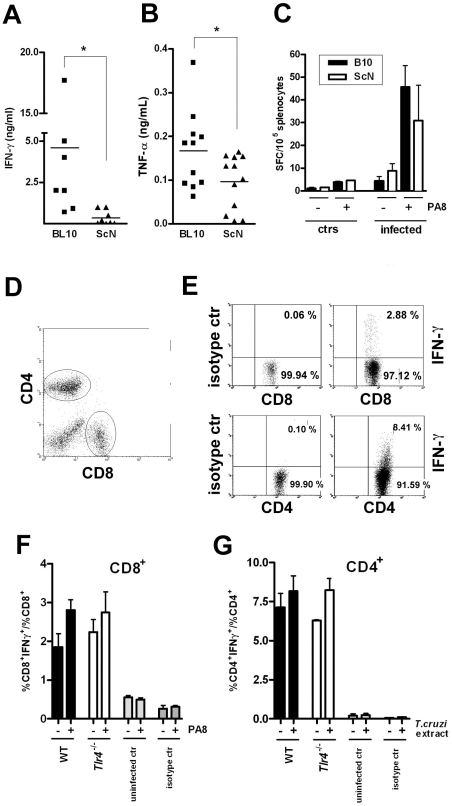
Preserved IFN-γ-producing CD8^+^and CD4^+^ T cell frequencies but reduced total IFN-γ and TNF-α levels in infected *Tlr4^−/−^* mice. Levels of cytokines (**A**) IFN-γ and (**B**) TNF-α, in the supernatant of splenocyte cultures from TLR4-deficient C57BL/10ScN mice (triangles) compared to the WT control, B10 mice (squares), at day 10 pi. Asterisk (*) indicates that the difference is statistically significant (p<0.05). (**C**) IFN-γ producing spleen cells specific to the PA8 peptide were estimated by the ELISPOT assay. Results obtained in C57BL/10ScN (white bars) and their wt controls B10 mice (black bars), uninfected or at day 20 pi. The results represent the mean number of peptide-specific spot forming cells (SFC) per 10^5^ splenocytes + SD (n = 4). (**D–G**) IFN-γ intracellular staining. (**D**) CD4 and CD8 dot plot gates. (**E**) Dot plot of IFN-γ staining and isotype controls of CD4^+^ or CD8^+^ spleen cells from WT infected mouse at day 13 pi, gated as in (**D**). (F and G) The ratio of IFN-γ^+^CD8^+^ cells within the CD8^+^ population (**F**) or of IFN-γ^+^CD4^+^ cells within the CD4^+^ population (**G**) was calculated for each mouse and condition: (−) unstimulated, or (+) stimulated with PA8 (**F**), or stimulated with *T. cruzi* extract (**G**), respectively. Results are expressed as the mean + SD of four to six animals per group. WT, black bars, T*lr4^−/−^*, white bars. Results of uninfected mice or isotype controls of both strains are shown in gray bars.

As IFN-γ is thought to be the most important inducer of iNOS in macrophages and thus essential for mediation of NO-dependent parasite control during acute infection [18], we quantified IFN-γ production by spleen cells from WT and TLR4-deficient infected mice. As shown in Figure 6A, higher IFN-γ levels are indeed secreted by WT infected splenocytes at day 10 pi. We also compared the secretion of another crucial cytokine for iNOS expression and host resistance to *T. cruzi*, TNF-α [14] As shown in Figure 6B, the levels of TNF-α secreted by splenocytes from infected WT mice are also significantly higher than from TLR4-deficient mice. Both IFN-γ and TNF-α can be produced after *T. cruzi*-induced triggering of the innate immune response (mainly by NK/NKT cells and macrophages/DC respectively), as well as by CD8^+^ and CD4^+^ T lymphocytes later in the infection course, as part of the acquired response to the parasite. Since several previous studies have demonstrated the importance of IFN-γ secretion by CD8^+^ T cells in resistance to infection with *T. cruzi* [19], we asked if the frequency of Ag-specific IFN-γ-secreting cells would be altered in the absence of TLR4 expression. To do so, a previously defined H-2K^b^-restricted epitope (PA8) derived from the amastigote surface protein-2 (ASP2), which is a member of the *trans*-sialidase family of surface proteins, was employed in ELISPOT assays [20]. However, as shown in Figure 6C no significant difference in the frequency Ag-specific IFN-γ secreting cells could be observed between WT and TLR4-deficient infected mice. Two other previously described *trans*-sialidase-derived peptides TSKB20 and TSKB18 [21] were alternatively employed in ELISPOT assays, giving the same results (not shown). The frequency of IFN-γ secreting CD4^+^ and CD8^+^ T cells in the spleens of WT and TLR4-deficient mice at day 13 pi was also investigated by intracellular staining and results are shown in Figure 6D-G. These data show that the frequencies of CD4^+^ and CD8^+^ T cells secreting IFN-γ in response to *T. cruzi*-derived antigens are not reduced in *Tlr4* ^−/−^ mice.

### The frequency of defined peptide/H-2K^b^-restricted IFN-γ secreting lymphocytes is not decreased in *Tlr2 ^−/^* ^−^, *Tlr4^−/−^*, *Tlr9^−/−^* or *Myd88^−/−^* infected mice, nor is cytotoxicity

**Figure 7:**
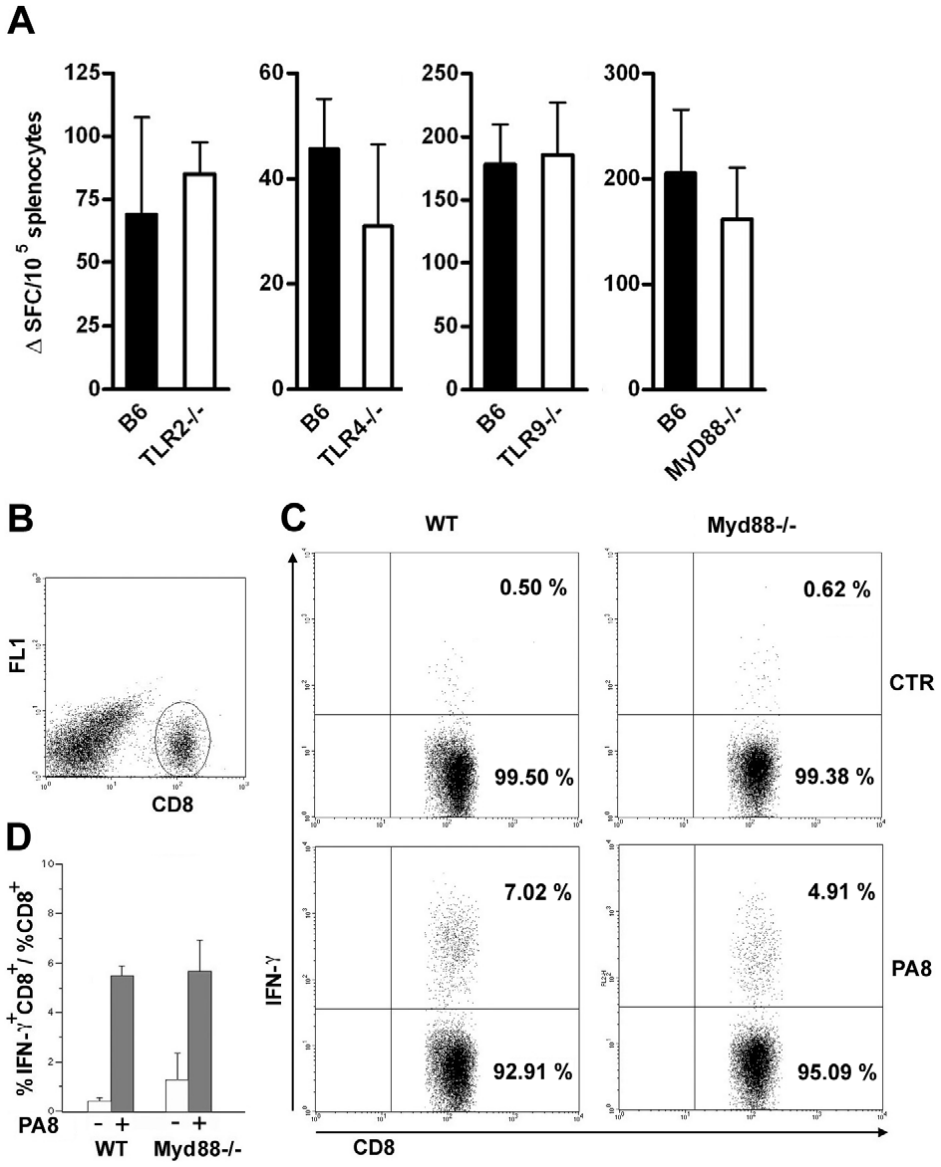
IFN-γ production by CD8^+^ T splenocytes is equivalent in infected *Myd88^−/−^*and WT mice. (**A**) IFN-γ producing spleen cells specific to the PA8 peptide were estimated by the ELISPOT assay in B6 (wt) and *Tlr2^−/−^*, *Tlr4^−/−^*, *Tlr9^−/−^*, or *Myd88^−/−^* mice, at days 15, 15, 15 and 10 pi, respectively. Values represent mean of ΔSFC (SFC obtained in the presence of PA8 peptide minus SFC obtained in the absence of peptide) per 10^5^ splenocytes + SD (n = 4 or 3). Results are representative of two or more independent experiments. IFN-γ production by CD8^+^ splenocytes from *T. cruzi-*infected (day 10 pi) *Myd88^−/−^* or WT mice was assessed following a 14-h *in vitro* incubation with or without PA8 peptide. After surface staining with PerCP-conjugated anti-CD8 mAb, the cells were fixed, permeabilized and then stained with PE-conjugated anti- IFN-γ mAb, and analyzed by flow cytometry. (**B**) Cells were gated on lymphocyte population (FSC × SSC) and CD8^+^ as shown. (**C**) CD8 versus IFN-γ dot-plot of gated cells: numbers indicate the percentage of IFN-γ-producing and non-producing CD8^+^ cells, within the CD8^+^ population. Data are representative of individual *Myd88^−/−^*and WT mice, stimulated with peptide (PA8) or not (ctr). (**D**) The ratio of IFN-γ^+^CD8^+^ cells within the CD8^+^ population was calculated for each mouse and condition (unstimulated, white bars or stimulated with PA8, gray bars) and results are expressed as the mean + SD of three to five animals per group.

**Figure 8:**
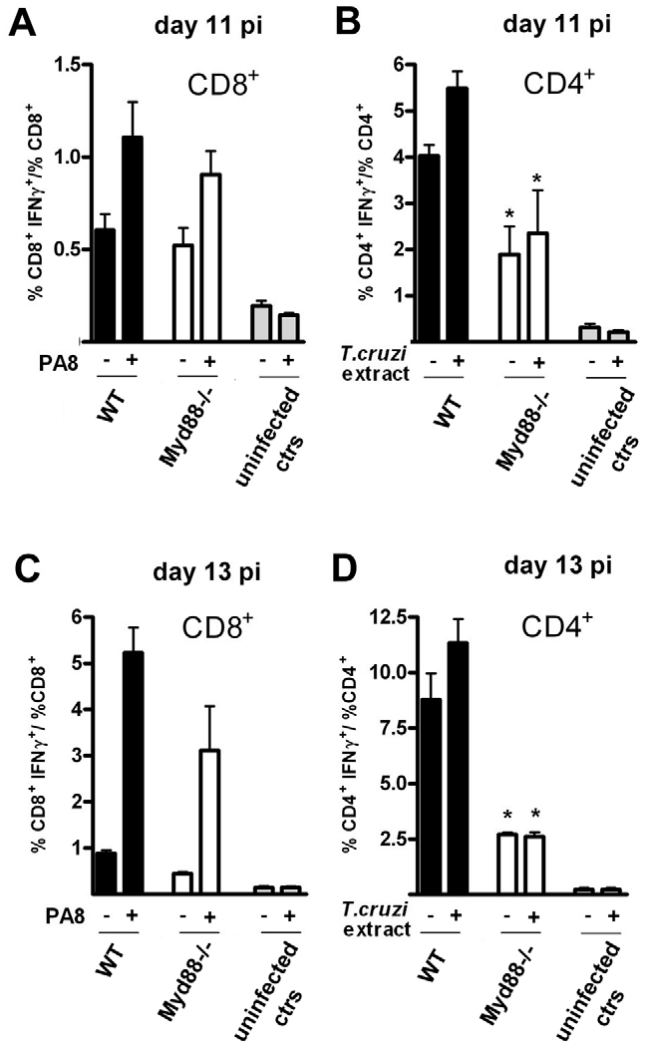
Preserved IFN-γ production by CD8^+^ T cells but impaired IFN-γ CD4^+^ response in *Myd88^−/−^* mice at days 11 and 13 pi. IFN-γ production by CD8^+^ (**A and C**) and CD4^+^ (**B and D**) splenocytes from *T. cruzi-*infected *Myd88^−/−^* or WT (B6) mice was assessed following a 5-h *in vitro* incubation with (+) or without (−) PA8 peptide (**A and C**) or *T. cruzi* extract (**B and D**), respectively, at days 11 (**A and B**) and 13 (**C and D**) pi. After surface staining with PerCP-conjugated anti-CD8 or FITC-conjugated anti-CD4 mAb, the cells were fixed, permeabilized and then stained with PE-conjugated anti-IFN-γ mAb, and analyzed by flow cytometry. Cells were gated on lymphocyte population (FSC×SSC) and CD8^+^ or CD4^+^ as shown in Fig. 6. The ratio of IFN-γ^+^CD8^+^ cells within the CD8^+^ population and the ratio of IFN-γ^+^CD4^+^ cells within the CD4^+^ population was calculated for each mouse and condition (WT, black bars, *Myd88^−/−^*, white bars) and results are expressed as the mean ± SD of four to six animals per group. Uninfected controls of both strains were pooled and shown in gray bars. Asterisk (*) indicates that the percentage of IFN-γ^+^CD4^+^ is significantly different (p<0.05) between WT and *Myd88^−/−^* mice, treated (+) or not (−) with *T. cruzi* extract.

**Figure 9:**
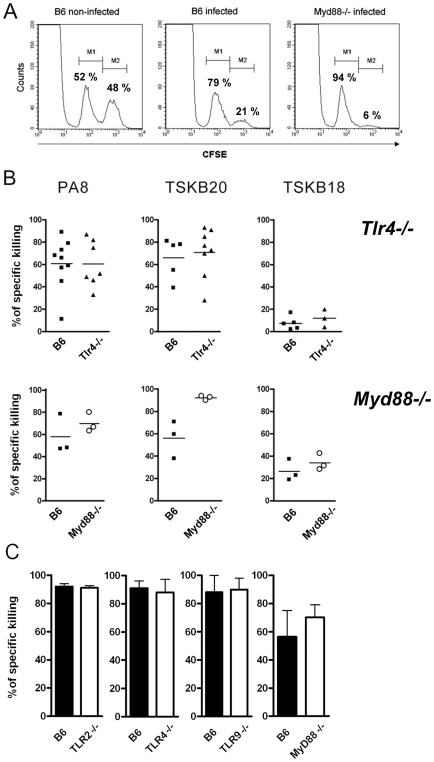
Specific cytotoxicity against immunodominant peptides in infected *Tlr^−/−^* or *Myd88^−/−^* mice. (**A**) *In vivo* cytotoxicity assays by cytometry. Normal splenocytes labeled with two concentrations of CFSE (CFSE^low^ and CFSE^high^) were injected intravenously into infected mice. CFSE^high^ cells (M2 population) were pulsed with the K^b^-restricted PA8 peptide derived from *T. cruzi* ASP-2 protein. CFSE^low^ cells (M1 population) were unpulsed and served as internal controls. Percentage of specific cell lysis was measured 20 hr later by FACS, as described in the Materials and Methods section. (**B**) The percentage of specific cell killing obtained *in vivo* in infected *Tlr4^−/−^* or *MyD88^−/−^* mice at days 20 and 10 pi, respectively, and in their WT control, B6 mice, using PA8-, TSKB20- or TSKB18-pulsed target cells. Results are expressed as the mean of three to nine animals per group, each symbol representing an individual mouse. (**C**) Percentage of specific *in vivo* cell killing obtained in *Tlr2^−/−^*, *Tlr4^−/−^*, *Tlr9^−/−^* or *MyD88^−/−^* mice (white bars) and in their wt control, B6 mice (black bars), using PA8-pulsed target cells at days 15, 15, 15 and 10 pi, respectively. Results are expressed as the mean + SD of three to five animals per group. Results are representative of three or more independent experiments.

At this point, the present investigation was extended to compare the frequency of PA8/K^b^-specific IFN-γ secreting lymphocytes between WT and mice which are deficient in other members of the TLR family, as well as *Myd88^−/−^* mice, whose susceptibility to *T. cruzi* infection was previously described [6], [7]. To our surprise, the frequency of these important effector cells of the acquired response is not altered in the spleens of *Tlr2^−/−^*, *Tlr9^−/^* ^−^ or *Myd88^−/−^* mice, as with *Tlr4*-deficient mice (Figure 7A). We also estimated the percentage of IFN-γ secreting CD8^+^ T cells in the spleen of infected *Myd88^−/−^* mice at day 10 pi by intracellular cytokine staining (ICS) following *in vitro* stimulation with PA8 peptide and obtained the same results, that is, no significant difference in the frequency of IFN-γ^+^CD8^+^cells between WT B6 and *Myd88^−/−^* mice (Fig. 7B–D). In order to further evaluate the adaptive response to *T. cruzi* in *Myd88^−/−^* mice, the percentage of IFN-γ secreting T cells was measured by ICS in both CD4^+^ and CD8^+^ subsets at days 11 and 13 pi. As shown in Figure 8, although the absence of MyD88 signaling strongly affects the percentage of CD4^+^ IFN-γ cells in these mice, MyD88 expression is not essential for the differentiation of IFN-γ producing CD8^+^ T cells specific against *T. cruzi*-derived epitopes. Finally, we tested whether expansion of specific CD8^+^ cytotoxic T cells was affected in any of the above *Tlr^−/−^* or *Myd88^−/−^* mice. For this purpose, we used a functional cytotoxic assay which measures the *in vivo* elimination of target cells (total splenocytes) coated either with PA8 (Figs. 9A and C), TSKB20 or TSKB18 (Fig. 9B) peptides, as previously described [21]. The phenotype of effector cells mediating peptide-specific *in vivo* cell killing was established earlier as being CD8^+^ T cells [22]. The kinetics of Ag-specific cytotoxic CD8^+^ T cell development during infection with the Y strain of *T. cruzi* in mice was also previously determined, showing that the maximum cytotoxicity (close to 100% specific lysis) is attained around day 15 pi and continued at a high level in B6 mice, even until 100 days after challenge [22]. As shown in Fig. 9B, at day 20 post-infection, no difference in peptide-specific cytotoxicity could be detected between *Tlr4^−/−^* and WT mice for any of the tested peptides. The same was true for *Myd88^−/−^* mice, in which the *in vivo* cytotoxicity assay was performed at an earlier post-infection time point (day 10 pi) due to their earlier mortality [7] (Fig. 9B). A summary of the cytotoxicity experiments is shown in Figure 9C, where the results of specific killing obtained with the PA8 immunodominant peptide in *Tlr2^−/−^*, *Tlr4 ^−/−^*, *Tlr9^−/−^* or *Myd88^−/−^* mice are compared to B6 controls. No difference in the levels of specific cytotoxicity was observed in any of these deficient mice. Together, these results clearly indicate that deficiency in TLR2, TLR4, TLR9 or even MyD88 expression does not impair CD8^+^ T cell effector responses during infection with *T. cruzi*.

## Discussion

Different *T. cruzi*-derived molecules are able to induce host innate immune responses through the activation of different members of the TLR family, [3]–[6], [23], including glycoinositolphospholipids derived from the parasite membrane which induce a pro-inflammatory response through the TLR4 pathway [5], [23]. Thus, the documented high susceptibility of *Myd88^−^* ^/−^ mice to infection with *T. cruzi* could not be attributed to a single TLR, suggesting that different members of the TLR family act in concert in determining resistance to the pathogen [6]. Bafica and collaborators have shown that doubly deficient *Tlr2^−/−^ Tlr9^−/−^* mice, although more susceptible than the single TLR2- or TLR9-deficient mice, do not display the acute mortality exhibited by *Myd88^−/−^* mice, suggesting that additional TLR/IL-1R family members are involved in the protection against infection with *T. cruzi* in mice [6]. In this context, the contribution of TLR4 signaling to control of the parasite burden in the C57BL/6 background was not investigated until the present study, as the only previous work on the subject was performed in mice of a different genetic background [5]. Importantly, the present work is the first to study the contribution of the different TLR and MyD88 pathways to the development of anti-*T. cruzi* responses mediated by CD8^+^ effector T cells, a critical element of the acquired immune response to the parasite.

The first question we addressed was to assess the role of TLR4 in *T. cruzi* internalization and triggering of very early microbicidal activity by macrophages. Infective *T. cruzi* trypomastigotes invade host cells using at least two different strategies; either by an active process recruiting host-cell lysosomes to the area of parasite cell contact or by an alternative pathway, in which the parasite infects phagocytic cells through conventional phagocytosis/endocytosis mechanism [24]–[26]. In both cases, the parasite may escape to the cytoplasm where it differentiates into the aflagellated amastigote form and begins intracellular replication. During cell invasion, *T. cruzi* interacts with different macrophage receptors to induce its own phagocytosis, but the nature of those receptors and the molecular mechanisms involved are still poorly understood. Although the general current view is that TLRs do not function directly as phagocytic receptors [27], a recent report indicated that during the invasion of *T. cruzi*, the activation of the Rab5-dependent phagocytic pathway is regulated by signals emanated through the parasite interaction with TLR2 in macrophages [28]. Our present results with *Tlr4*-deficient macrophages from three different mouse strains show that internalization of *T. cruzi* by macrophages is not affected by the absence of functional TLR4 expression. Some studies on the other hand, have demonstrated that TLR signaling by means of MyD88 can enhance phagosome acidification and function, the so-called phagosome maturation, which is required for effective sterilization of its contents [29]. In accord with those results, we found that after 2.5 h of infection, TLR4 and parasite co-localize into acidic compartments. Also, 4 h after infection, the percentage of TLR4-deficient macrophages infected with *T. cruzi* is significantly higher when compared to WT cells. The addition of iNOS or ROS inhibitors abolished the difference in the frequency of infected macrophages between cultures from TLR4-deficient and WT origin, indicating that this early trypanosomicidal mechanism triggered by TLR4 depends on the production of reactive nitrogen intermediates (RNI) and ROS, which have been described to participate in the microbicidal activity against *T. cruzi* and other pathogens [12]–[15]. Moreover, the fact that the simultaneous usage of iNOS and ROS inhibitors did not increase further the percentage of infected macrophages, suggests that the peroxynitrite anion (ONOO^−^), a strong oxidizing and against *T. cruzi*, formed by the reaction between nitric oxide (NO) and superoxide radical (O^−^_2_), may be the main species responsible for the elimination of *T. cruzi*, as described [30], [31]cytotoxic effector molecule. Therefore, TLR4 signaling triggers an important early parasiticidal event against *T. cruzi*, which is dependent on the formation of NO and ROS. Significantly lower production of NO was also found in splenocyte cultures from *Tlr4* ^−/−^ mice at day 10 post infection. In conformity to these results, we demonstrated that *Tlr4* ^−/−^ splenocyte cultures produce lower levels of the main iNOS inducer cytokines, IFN-γ and TNF-α. As with the *in vitro* results obtained with macrophage cultures, the inhibition of NO production during *in vivo* infection made WT and *Tlr4* ^−/−^ mice equally susceptible, as measured by mortality and parasite levels in the blood. Our results are in agreement with previous studies demonstrating that mice deficient for inducible nitric oxide synthase (iNOS) are highly susceptible to *T. cruzi* [32], and that the inhibition of iNOS from the beginning of infection lead to an increase in trypomastigotes in the blood and to high mortality [15], [33]. Together, our results point to a significant contribution of the TLR4 pathway to the innate immune response against *T. cruzi* infection, with the production of NO playing a major role.

We show that mice of either B10 or B6 genetic background with TLR4 deficiency presented significantly elevated parasite numbers in the blood compared to their WT controls after *in vivo* infection with *T. cruzi*. These results are in accordance with our previous work showing higher parasitemia levels of the *Tlr4* mutant C3H/HeJ mice [5], although this is more pronounced in the latter lineage. Concerning mortality, however, the absence of functional TLR4 expression in B10 or B6 mice do not lead to the acute mortality previously observed in C3H/HeJ mice [5]. Therefore, the effects of TLR4 deficiency on susceptibility to infection with *T. cruzi* are more evident in the C3H background. Inbred strains of mice may vary from highly resistant to highly susceptible, as reflected by parasitemia levels and survival time and, following these criteria, C3H strains have been classified as “susceptible”. Classical genetic studies previously established that the resistance to *T. cruzi* is governed by multiple genetic factors, including *H-2*-linked gene(s) [34], [35] and the combination of different alleles in a group of loci confers resistance or susceptibility to infection. Therefore, analogous to the effects due to the absence of TLR2, which only become perceptible in mice with the concomitant deficiency on TLR9 [6], we have shown here that the susceptibility resulting from the absence of TLR4 is less pronounced in the resistant B6 and B10 backgrounds, compared to C3H strains.

IFN-γ is an important mediator of resistance to *T. cruzi*. Besides iNOS, IFN-γ regulates the expression of a large number of genes, including chemokines and chemokine receptors, which were shown to play a role in IFN-γ-mediated protection in *T. cruzi* infection [18], [32], [36]. Early during infection, IFN-γ is secreted by NK cells and other cell types, as part of the innate response, and later on the infection course by activated CD4^+^ and CD8^+^ T cells. Since TLRs have been implicated in the modulation of acquired immunity against several pathogens, we have herein addressed the question of whether the frequencies of IFN-γ secreting CD8^+^ and CD4^+^ T cells are altered in *Tlr4^−/^* mice. Our data showed no significant difference in the frequencies of IFN-γ producing CD8^+^ or CD4^+^ T cells in the spleens of *Tlr4* ^−/−^ and WT mice, indicating that TLR4 deficiency does not interfere with these important effectors of the acquired response against *T. cruzi*. Therefore, the higher level of IFN-γ detected in the supernatants of WT splenocyte cultures at day 10 pi is probably contributed by cells of the innate response. A number of different cells types may account for that and we are currently evaluating their phenotype.

On the other hand, a significant reduction in the levels of the IFN-γ^+^CD4^+^ T cell population was observed in the spleens of *Myd88^−/−^* infected mice. This finding is in line with previous *in vitro* experiments [6], but contrasts to the results of a recent paper, where IFN-γ production by CD4^+^ T cells is shown to be preserved in *Myd88^−/−^* mice infected with the Tulahuen strain of *T. cruzi* [37]. The reason for this apparent discrepancy is not clear and could be due to the different strain of parasite used for infection or to the method employed for CD4^+^ T cell re-stimulation *in vitro*. Therefore, our results demonstrate that although the CD4^+^ T cell-mediated response is substantially diminished, unaltered frequencies of CD8^+^ IFN-γ T cells specific for K^b^-restricted *T. cruzi*-derived peptides are present in the spleens of *Myd88^−/−^* mice compared to WT controls at days 10, 11 and 13 pi.

CD8^+^ T cell mediated responses are a critical component of protective immunity in *T. cruzi* infection, since in their absence mice quickly succumb to the infection or develop a more severe chronic disease (reviewed in [19]). Moreover, CD8^+^ T cells can be induced by vaccination to provide protection from lethal infection [38]. CD8^+^ T cells can control infection via a number of mechanisms: in addition to the already discussed secretion of IFN-γ inducing microbicidal activity in the host cell, the direct cytotoxic function against cells infected with *T. cruzi* is also a main effector response. Therefore, it is an important issue to define whether TLR-MyD88 mediated pathways can play a role in the priming and/or control of the cytotoxic T cell response against *T. cruzi*-infected targets. According to the present paradigm, this could be mainly achieved by the engagement of TLRs on antigen-presenting cells (APCs) such as dendritic cells (DCs), promoting upregulation of co-stimulatory molecules, enhancement of antigen processing and presentation, as well as secretion of Th1 polarizing pro-inflammatory cytokines by the DCs [2]. We demonstrated here, however, that the cytotoxic response mediated by CD8^+^ T cells against H-2K^b^-restricted immunodominant peptides in *T. cruzi* infected mice is not dependent on TLR2, TLR4 nor TLR9 expression. Unexpectedly, the Ag-specific cytotoxic function was also preserved in *Myd88^−/−^* mice. As the cytotoxic response against the immunodominant PA8 *T. cruzi* epitope tested here was previously shown to be dependent on MHC class II restricted CD4^+^ T cells [39], our results indicate that although diminished in frequency, the residual response of CD4^+^ activated T cells observed in infected *Myd88^−/−^* mice is sufficient for their licensing function, which results in the development of parasite-specific CD8^+^mediated cytotoxic response. A first possible interpretation of these results is that none of the tested TLR and MyD88 pathways are involved in the generation of cytotoxic CD8^+^ T cells during *T. cruzi* infection. In fact, other signaling molecules and innate recognition systems might contribute to adaptive immunity to *T. cruzi* as the members of the Nod-like receptor protein (NLR) family [40]. Other examples are: 1) the release of pro-inflammatory bradykinin peptide by the parasite proteases during infection and consequent DC maturation induced by bradykinin B2 receptors (B2R) [41] and 2) the recently described DC maturation induced by NFATc1 activation and consequent IFN-γ production in a TLR-independent pathway [37]. However, to date, it was not determined if these TLR-independent pathways can fully account for the preserved CD8^+^ T cell cytotoxic response against *T. cruzi*-infected targets observed in *Myd88^−/−^* mice.

In our opinion, there is another plausible hypothesis to be considered for explaining the preserved CD8^+^ T cell cytotoxic response in *Tlr2^−/−^*, *Tlr4^−/−^*, *Tlr9^−/−^* or *Myd88^−/−^* mice: it is known that type I IFNs affect DC maturation [42], [43] and can also stimulate survival, development of cytolytic function, and production of IFN-γ by CD8^+^ T cells [44], [45]. Moreover, mice deprived of the type I IFN receptor, *Ifnar* ^−/−^, develop higher parasitemia levels in comparison with control 129Sv mice [46] and doubly deficient *Myd88^−/−^Ifnar^−/−^* mice are highly susceptible to infection with *T. cruzi* [8]. Both TLR9 and TLR4 could induce type I IFN secretion through MyD88-dependent and -independent pathways, respectively. Therefore, TLR4 and TLR9 would be redundant concerning type I IFN production and might compensate for each other's absence in *Tlr4^−/−^* or *Tlr9^−/−^* mice. The TLR4-triggered TRIF pathway is also preserved in *Myd88^−/−^* mice and its activation would lead to type I IFN secretion and DC maturation, with the consequent normal adaptive responses against *T. cruzi* in these mice. Testing whether the CD8-mediated cytotoxicity against *T. cruzi* is affected in *Tlr4^−/−^Tlr9^−/−^ or Tlr4^−/−^Myd88^−/−^* doubly deficient mice is one of our future goals. According to this hypothesis, cytotoxic CD8^+^ T cells would not be preserved in doubly deficient *Myd88^−/−^Trif^−/−^* mice, which is in agreement with the fact that these mice are even more susceptible to infection, as indicated by accelerated mortality when compared to single *Myd88^−/−^* mice [8]. Also, in opposition to *Myd88^−/−^*, the doubly deficient *Myd88^−/−^Trif^−/−^* mice are not able to control the levels of parasite in the blood [8].

The maintenance of CD8^+^ acquired responses against *T. cruzi* in *Myd88^−/−^* mice finds a parallel in studies of murine infection with *Toxoplasma gondii*, another intracellular protozoan parasite. As for *T. cruzi*, multiple TLR ligands were identified in *T. gondii* and *Myd88^−/−^* mice were shown to be highly susceptible to infection (reviewed in [47]). Interestingly, a recent work demonstrated that a robust and protective IFN-γ response can be elicited in *Myd88^−/−^* mice infected with an avirulent *T. gondii* strain [48]. Therefore, the MyD88 pathway is required for innate immunity to control infection with *Toxoplasma*, even though adaptive immunity against the pathogen can be triggered without the need for this TLR adaptor molecule. The same picture emerges from our present results with *T. cruzi*. The absence of a role for the MyD88 pathway in the generation of CD8^+^ adaptive responses during *T. cruz*i infection is also in line with other previous reports analyzing the immune response to other pathogens [49], [50], including the described protective CD8^+^ T cell response against the intracellular bacteria *Listeria* [51].

The preservation of CD8^+^ T cell mediated effector mechanisms in MyD88-deficient mice is in agreement with the fact that despite their high mortality, these mice do succeed in controlling the number of parasites in the blood, in contrast to the even more susceptible *Ifng^−/−^*, *IfngR^−/−^*, *iNos^−/−^* or *Myd88^−/−^Trif^−/−^* mice [8], [32], [36]. At present we do not know why *Myd88^−/−^*mice succumb earlier than WT mice and display 100% mortality to *T. cruzi* infection, notwithstanding their capacity of controlling blood parasitemia [7] and their preserved CD8^+^ T cell-mediated responses shown here. We would like to consider four non-exclusive possibilities: First, *Myd88^−/−^*mice, whose IFN-γ levels in serum were shown to be significantly lower [7], would also be affected by the fact that several genes, like iNOS and IP-10, have been shown to be 5- to 100-fold less extensively induced by IFN-γ in macrophages lacking MyD88 expression [52]. Second, the higher susceptibility of *Myd88^−/−^* mice could be directly attributed to the defective activation of CD4^+^ T cells demonstrated here, as this cell population has also been demonstrated to be essential for resistance to infection [53], probably through IFN-γ and TNF-α secretion. We do not know at the present what mechanism, absent in *Myd88^−/−^* mice, affects CD4^+^ T cell activation. Both attenuated DC maturation due to the absence of TLRs/MyD88 triggering and the absence of IL-1R/IL-18R signaling in CD4^+^ *Myd88^−/−^* T cells should be considered. Third, the lower levels of CD4^+^ helpers might also have indirect consequences as a defect in the B cell mediated response, which was also described to be necessary for resistance to the parasite [54]. In fact, although controversy exists, the requirement of TLR-MyD88 signaling for the generation of T-dependent antigen-specific antibody responses was proposed [55], [56] and, interestingly, antibody responses against different virus are altered or completely lost in *Myd88^−/−^* mice [57]–[59]. Finally, another possible consequence of a deficiency in CD4^+^ cell activation could be the defective migration of CTLs into peripheral sites of infection distinct of the spleen (liver and heart, for example), as recently demonstrated by Nakanishi et al. in a mouse model of herpes simplex virus (HSV) infection of the vagina [60].

In summary, the results obtained in the present study strongly argue in favor of a role for the TLR4 signaling in the innate immune response against *T. cruzi* displayed by B6 mice. Notably, we have also shown here that neither the absence of TLR2, TLR4 or TLR9 individually, nor the ablation of all MyD88-mediated pathways affect the development of cytotoxic and IFN-γ-producing CD8^+^ T cells, which are crucial effector mechanisms against this parasite. Determining precisely how TLR-TRIF-MyD88 activation contributes to trigger protective immunity against *T. cruzi* will be of critical relevance for vaccine development against this important human parasite.

## Materials and Methods

### Mice and ethics statement

All animal experiments were approved by and conducted in accordance with guidelines of the Animal Care and Use Committee of the Federal University of Rio de Janeiro (Comitê de Ética do Centro de Ciências da Saúde CEUA -CCS/UFRJ). *Tlr2^−/−^*, *Tlr4^−/−^*, *Tlr9^−/−^* and *Myd88^−/−^* mice were generated by and obtained from Dr. S. Akira (Osaka University, Japan) via Dr. R. T. Gazzinelli (Federal University of Minas Gerais, Brazil). *Tlr2^−/−^* and *Tlr4^−/−^* mice were maintained along C57BL/6 mice at the Laboratório de Animais Transgênicos (LAT, IBCCF°, UFRJ, RJ, Brazil). C3H/HeJ and C3H/HePas mice were from ICB, Universidade de São Paulo (USP, SP, Brazil). C57BL/10 and C57BL/10ScN mice were maintained at the Biotério of the Department of Immunology (IMPPG, UFRJ, RJ, Brazil). *Tlr9^−/−^* and *Myd88^−/−^* mice were maintained at the Centro de Pesquisas René Rachou (FIOCRUZ, MG, Brazil).

### Parasite and experimental infection

Mice used for experiments were sex- and age-matched, and housed with a 12-h light-dark cycle. Bloodstream trypomastigotes of the Y strain of *T. cruzi* [61] were obtained from BALB/c mice infected 7 days earlier. The concentration of parasites was estimated and each mouse (at least 4 per group) was inoculated intraperitoneally (i.p.) with 0.2 ml (2×10^3^ trypomastigotes). Parasitemia was monitored by counting the number of bloodstream trypomastigotes in 5 µl of fresh blood collected from the tail vein. Mouse survival was followed daily.

### In vitro infection

Resident or elicited macrophages (obtained from the peritoneal cavity on day 4 after injection of 2.5 ml of 3% thioglycollate) were plated in triplicates and infected with trypomastigotes at a 1∶10 (macrophage:trypomastigote) ratio. After 1 h of infection, the cells were washed four times with PBS to remove the extracellular parasites and cultured in DMEM supplemented with 10% FBS (GIBCO, Invitrogen) for the indicated time periods at 37°C in an atmosphere containing 5% CO_2_. Trypomastigotes in the culture supernatants were counted microscopically in triplicates. Alternatively, extracellular parasites were removed by repeated washing after 1 h of infection and the cells were either washed, fixed and stained with Giemsa or cultured for a further 4 h in DMEM supplemented with 10% FBS before fixation and staining. In other experiments, macrophages were infected for 1 h in the presence of L-NMMA (1 mM) and/or DFO (100 µM); after washing, cells were cultured for further 4 h in DMEM supplemented with 10% FBS in the presence of L-NMMA (1 mM) and/or DFO (100 µM) and subsequently fixed and stained with Giemsa. The percentage of infected macrophages and the intracellular parasite numbers in 100 macrophages were counted under a light microscope.

### Confocal microscopy

The stable cell line of HEK293 cells expressing the fluorescent protein TLR4^YFP^ and MD-2 constructs were described previously [11] and kindly donated by Dr D. Golenbock (University of Massachusetts Medical School, MA). Trypomastigotes were labeled with TO-PRO-3 (1 µl/ml, Molecular Probes, Invitrogen) for 30 min at RT, washed twice and then cultured with HEK- TLR4^YFP^ cells at 10∶1 ratio for 1 hour at 37°C, 5% CO_2_. After repeated washing with PBS for extracellular parasite removal, cells were stained with LysoTracker Red probe (75 nM, Molecular Probes, Invitrogen) for 1 hour. Cells were then washed in PBS containing 1 mM MgCl2 and 1 mM CaCl2 and fixed in 3,7% paraformaldehyde/PBS for 15 min. Confocal microscopy was performed with a Zeiss Axiovert 200-M inverted microscope equipped with an LSM 510 Meta laser-scanning unit. Image analysis was performed with LSM 510 software (Zeiss).

### Griess reaction for RNI quantification

The Griess reaction was performed to quantitate nitrite concentrations in the supernatant of macrophage or spleen cell cultures, as previously described [62]. Briefly, 50 µl of sample plus 50 µl of Griess reagent were incubated for 10 min at RT, followed by detection at 550 nm in an automated ELISA plate reader. The results are expressed in units of micromolar, and were determined comparing the absorbance readings of the experimental samples to a sodium nitrate standard curve.

### Inhibition of iNOS *in vivo*

Inhibition of iNOS *in vivo* was performed by injecting mice i.p. with 50 mg/kg of aminoguanidine/body weight, (AG, Sigma-Aldrich, St. Louis), diluted in sterile phosphate-buffered saline (PBS), every other day, as previously described [20]. Treatment started 4 h before infection with *T. cruzi* (performed as described above), and animals were treated until day 13 pi. Control mice received the same volume (200 µl) of PBS. A third group of mice received AG only.

### ELISPOT

The ELISPOT assay was performed essentially as described earlier [63]. Briefly, the preparation of plates was done by coating 96-well nitrocellulose plates Multiscreen HA (Millipore) with 60 µl/well of sterile PBS containing 10 µg/ml of the anti-mouse IFN-γ mAb R4-6A2 (BD Biosciences, San Jose, CA). After overnight incubation at RT, mAb solution was removed by sterile aspiration and the plates were washed three times with plain RPMI 1640 medium under sterile conditions. Plates were blocked by incubating wells with 100 µl RPMI medium containing 10% (v/v) FBS for at least 2 h at 37°C. Responder cells were obtained from spleens of B6 mice. Responder cells were ressuspended to a concentration of 10^6^ viable cells per ml in RPMI medium (GIBCO, Invitrogen) supplemented with 10 mM HEPES, 2 mM L-glutamine, 5×10^−5^ M 2-β-mercaptoethanol, 1 mM sodium pyruvate, 100 U/ml of penicillin and streptomycin, 10% (v/v) FBS (all purchased from GIBCO, Invitrogen). B6 spleen cells adjusted to a concentration of 4×10^6^ viable cells per ml were used as antigen presenting cells after incubation or not with the synthetic peptide at a final concentration of 10 µM for 30 min at 37°C. One hundred microliters of suspension containing responders or antigen presenting cells were pipetted into each well. The plates were incubated for 24 h at 37°C in an atmosphere containing 5% CO_2_. After incubation, the bulk of cultured cells was flicked out. To remove residual cells, plates were washed 3 times with PBS and 3 times with PBS-Tween. Each well received 75 µl of biotinylated anti-mouse IFN-γ mAb XMG1.2 (BD Biosciences) diluted in PBS-Tween to a final concentration of 2 µg/ml. Plates were incubated overnight at 4°C. Unbound antibodies were removed by washing the plates at least 6 times with PBS-Tween. Peroxidase-labeled streptavidin (KPL) was added at a 1∶800 dilution in PBS-Tween in a final volume of 100 µl/well. Plates were incubated for 1–2 h at RT and then washed three to five times with PBS-Tween and three times with PBS. Plates were developed by adding 100 µl/well of peroxidase substrate (50 mM Tris–HCl at pH 7.5 containing 1 mg/ml of DAB and 1 µl/ml of 30% hydrogen peroxide solution, both from Sigma). After incubation at RT for 15 min, the reaction was stopped by discarding the substrate solution and rinsing the plates under running tap water. Plates were dried at RT and spots were counted with the aid of a stereomicroscope (Nikon) or in the ImmunoSpot® Analyzer (Cellular Technology Ltd., Shaker Heights, OH, USA). Results of the ELISPOT assay are representative of two or more independent experiments.

### Generation of *T. cruzi* amastigote extract

Tissue culture trypomastigotes of the Y strain of *T. cruzi* were transformed to amastigotes in acidic DMEM/10% FCS for 24 h at 37°C, as previously described [64]. Parasites were pelleted, washed in PBS, and subjected to more than five rounds of freeze-thawing followed by sonication. Cellular debris were removed by centrifugation at 12,000 rpm, and the soluble fraction was boiled for 5 min to denature the proteins. Protein concentrations were determined using a Bio-Rad protein assay.

### Intracellular cytokine staining

Splenocytes isolated from infected mice were cultured either with *T. cruzi* amastigote extract at 10 µg/ml (see above) or with PA8 peptide (VNHRFTLV) at 10 µM, or left unstimulated, for 5 h to 14 h at 37°C in the presence of brefeldin A (Sigma-Aldrich). Cells were surface stained with anti-CD8-PerCP and anti-CD4-FITC (BD Biosciences) and fixed for 10 minutes with a solution containing PBS, 4% paraformaldehyde at RT. Then, cells were permeabilized for 15 minutes with PBS, 0.1% bovine serum albumine, 0.1% saponin (Sigma-Aldrich). Intracellular cytokine staining was performed with anti- IFN-γ -PE (BD Biosciences). At least 10,000 gated CD8^+^ lymphocyte events were acquired. Analytical flow cytometry was conducted with a FACSCalibur (BD Biosciences) and the data were processed with CellQuest software (BD Biosciences).

### *In vivo* cytotoxicity assay

For the in vivo cytotoxicity assays, splenocytes of the different mouse strains were divided into two populations and labeled with the fluorogenic dye CFSE (Molecular Probes, Invitrogen) at a final concentration of 5 µM (CFSE^high^) or 0.5 µM (CFSE^low^). CFSE^high^ cells were pulsed for 40 min at 37°C with 1–2.5 µM of either H-2K^b^ -restricted ASP-2 peptide, also called PA8, (VNHRFTLV), H-2K^b^- restricted TsKb-18 peptide (ANYDFTLV) or H-2K^b^- restricted TsKb-20 peptide (ANYKFTLV). CFSE^low^ cells remained unpulsed. Subsequently, CFSE^high^ cells were washed and mixed with equal numbers of CFSE^low^ cells before injecting i.v. 15–20×10^6^ total cells per mouse. Recipient animals were mice that had been infected or not with *T. cruzi*. Spleen cells of recipient mice were collected 20 h after transfer, fixed with 2.0% paraformaldehyde and analyzed by cytometry, using a FACSCalibur Cytometer (BD Biosciences). Percentage of CFSE^low^ (M1) and CFSE^high^ (M2) cells were obtained using CellQuest software (BD Biosciences). Percentage of specific lysis was determined using the formula: 1 - ((M2infected/M1infected)/(M2naïve/M1naive)) ×100%.

### Statistical analysis

Statistical analyses were performed using GraphPad Prism version 4.00 for Windows (GraphPad Software, San Diego California USA, www.graphpad.com). Data were compared using a two-tailed Student's *t* test and are expressed as mean ± SEM. Data were considered statistically significant if *p* values were <0.05. The LogRank test was used to compare the mouse survival rate after challenge with *T. cruzi*. The differences were considered significant when the *P* value was <0.05.

## Supporting Information

Figure S1 Early trypanosomacidal mechanism is absent in TLR4-deficient macrophages. **(A)** Resident macrophages from C3H/HePas (wt, black bars) and C3H/HeJ (*Tlr4^d/d^*, white bars) mice were infected with blood form trypomastigotes of the Y strain in a 1∶10 (macrophage:trypomastigotes) ratio, for 1 h. After this period, extracellular trypomastigotes were removed by washing and the cells were fixed and stained with Giemsa (1 h). Alternatively, after washing, cultures were prolonged for a total 4-h incubation time, after which cells were fixed and stained with Giemsa (4 h). **(B)** Resident macrophages from C57BL/10 (wt, black bars) and C57BL/10ScN (*Tlr4^−/−^*, white bars) were infected and treated as in **(A)**. The percentage of infected macrophages was counted under a light microscope and each data point is expressed as the mean + SEM of triplicates. Experiments shown are representative of at least two independent experiments. One asterisk (*) indicates that the percentage of infected MO is significantly different (p<0.05) between WT and TLR4-deficient MO after the 4 h period of culture. Two asterisks (**) indicate that the percentage of infected MO is significantly different (p<0.05) between WT MO cultivated by 1 h and 4 h periods of culture.
(0.61 MB TIF)

Figure S2 IFN-γturns WT and *Tlr4^−/−^* MO equally resistant to infection with *T. cruzi*. 10^5^ resident peritoneal macrophages from *Tlr4^−/−^* mice and from their WT controls (C57BL/6) were cultured in the presence of trypomastigotes of the Y strain in a 1∶10 (macrophage:trypomastigotes) ratio, for 1 h. After removal of extracellular trypomastigotes by extensive washing, cultures were continued for several days and the number of trypomastigotes released into the supernatants was determined daily from day 10 on. In some triplicates, rmIFN-γ (2.0 ng/ml) was added to the cultures from the beginning (black symbols). Asterisks (*) indicate that the number of trypomastigotes released into the supernatants is significantly different (p<0.05) between WT and TLR4-deficient MO cultures. Each data point is expressed as the mean ± SEM of triplicates and experiments shown are representative of at least two independent experiments.
(0.76 MB TIF)

## References

[1] Dutra WO, Gollob KJ (2008). Current concepts in immunoregulation and pathology of human Chagas disease.. Curr Opin Infect Dis.

[2] Iwasaki A, Medzhitov R (2004). Toll-like receptor control of the adaptive immune responses.. Nat Immunol.

[3] Campos MA, Almeida IC, Takeuchi O, Akira S, Valente EP (2001). Activation of Toll-like receptor-2 by glycosylphosphatidylinositol anchors from a protozoan parasite.. J Immunol.

[4] Ouaissi A, Guilvard E, Delneste Y, Caron G, Magistrelli G (2002). The *Trypanosoma cruzi* Tc52-released protein induces human dendritic cell maturation, signals via Toll-like receptor 2, and confers protection against lethal infection.. J Immunol.

[5] Oliveira A-C, Peixoto RJ, Arruda LB, Campos MA, Gazzinelli RT (2004). Expression of functional TLR4 confers pro-inflammatory responsiveness to *Trypanosoma cruzi* glycoinositolphospholipids and higher resistance to infection with T. cruzi.. J Immunol.

[6] Bafica A, Santiago HC, Goldszmid R, Ropert C, Gazzinelli RT (2006). Cutting edge: TLR9 and TLR2 signaling together account for MyD88-dependent control of parasitemia in *Trypanosoma cruzi* infection.. J Immunol.

[7] Campos MA, Closel M, Valente EP, Cardoso JE, Akira S (2004). Impaired production of proinflammatory cytokines and host resistance to acute infection with *Trypanosoma cruzi* in mice lacking functional myeloid differentiation factor 88.. J Immunol.

[8] Koga R, Hamano S, Kuwata H, Atarashi K, Ogawa M (2006). TLR-dependent induction of IFN-β mediates host defense against *Trypanosoma cruzi*.. J Immunol.

[9] Poltorak A, He X, Smirnova I, Liu MY, Van Huffel C (1998). Defective LPS signaling in C3H/HeJ and C57BL/10ScCr mice: mutations in Tlr4 gene.. Science.

[10] Takeuchi O, Hoshino K, Kawai T, Sanjo H, Takada H (1999). Differential roles of TLR2 and TLR4 in recognition of gram-negative and gram-positive bacterial cell wall components.. Immunity.

[11] Latz E, Visintin A, Lien E, Fitzgerald KA, Monks BG (2002). Lipopolysaccharide rapidly traffics to and from the Golgi apparatus with the toll-like receptor 4-MD-2-CD14 complex in a process that is distinct from the initiation of signal transduction.. J Biol Chem.

[12] Tanaka Y, Kiyotaki C, Tanowitz H, Bloom BR (1982). Reconstitution of a variant macrophage cell line defective in oxygen metabolism with a H2O2-generating system.. Proc Natl Acad Sci U S A.

[13] Locksley RM, Klebanoff SJ (1983). Oxygen-dependent microbicidal systems of phagocytes and host defense against intracellular protozoa.. J Cell Biochem.

[14] Munoz-Fernandez MA, Fernandez MA, Fresno M (1992). Synergism between tumor necrosis factor-alpha and interferon-gamma on macrophage activation for the killing of intracellular *Trypanosoma cruzi* through a nitric oxide-dependent mechanism.. Eur J Immunol.

[15] Vespa GN, Cunha FQ, Silva JS (1994). Nitric oxide is involved in control of *Trypanosoma cruzi*-induced parasitemia and directly kills the parasite in vitro.. Infect Immun.

[16] Balcerczyk A, Sowa K, Bartosz G (2007). Metal chelators react also with reactive oxygen and nitrogen species.. Biochem Biophys Res Commun.

[17] Martins GA, Vieira LQ, Cunha FQ, Silva JS (1999). Gamma interferon modulates CD95 (Fas) and CD95 ligand (Fas-L) expression and nitric oxide-induced apoptosis during the acute phase of Trypanosoma cruzi infection: a possible role in immune response control.. Infect Immun.

[18] Gazzinelli RT, Oswald IP, Hieny S, James SL, Sher A (1992). The microbicidal activity of interferon-gamma-treated macrophages against Trypanosoma cruzi involves an L-arginine-dependent, nitrogen oxide-mediated mechanism inhibitable by interleukin-10 and transforming growth factor-beta.. Eur J Immunol.

[19] Martin D, Tarleton R (2004). Generation, specificity, and function of CD8+ T cells in *Trypanosoma cruzi* infection.. Immunol Rev.

[20] Low HP, Santos MA, Wizel B, Tarleton RL (1998). Amastigote surface proteins of *Trypanosoma cruzi* are targets for CD8+ CTL.. J Immunol.

[21] Martin DL, Weatherly DB, Laucella SA, Cabinian MA, Crim MT (2006). CD8+ T-Cell responses to *Trypanosoma cruzi* are highly focused on strain-variant trans-sialidase epitopes.. PLoS Pathog.

[22] Tzelepis F, de Alencar BC, Penido ML, Gazzinelli RT, Persechini PM (2006). Distinct kinetics of effector CD8+ cytotoxic T cells after infection with *Trypanosoma cruzi* in naive or vaccinated mice.. Infect Immun.

[23] Medeiros MM, Peixoto JR, Oliveira AC, Cardilo-Reis L, Koatz VL (2007). Toll-like receptor 4 (TLR4)-dependent proinflammatory and immunomodulatory properties of the glycoinositolphospholipid (GIPL) from *Trypanosoma cruzi*.. J Leukoc Biol.

[24] Tardieux I, Webster P, Ravesloot J, Boron W, Lunn JA (1992). Lysosome recruitment and fusion are early events required for trypanosome invasion of mammalian cells.. Cell.

[25] Burleigh BA (2005). Host cell signaling and *Trypanosoma cruzi* invasion: do all roads lead to lysosomes?. Sci STKE.

[26] Woolsey AM, Sunwoo L, Petersen CA, Brachmann SM, Cantley LC (2003). Novel PI 3-kinase-dependent mechanisms of trypanosome invasion and vacuole maturation.. J Cell Sci.

[27] Underhill DM, Gantner B (2004). Integration of Toll-like receptor and phagocytic signaling for tailored immunity.. Microbes Infect.

[28] Maganto-Garcia E, Punzon C, Terhorst C, Fresno M (2008). Rab5 activation by Toll-like receptor 2 is required for *Trypanosoma cruzi* internalization and replication in macrophages.. Traffic.

[29] Blander JM, Medzhitov R (2004). Regulation of phagosome maturation by signals from toll-like receptors.. Science.

[30] Denicola A, Rubbo H, Rodriguez D, Radi R (1993). Peroxynitrite-mediated cytotoxicity to *Trypanosoma cruzi*.. Arch Biochem Biophys.

[31] Alvarez MN, Piacenza L, Irigoin F, Peluffo G, Radi R (2004). Macrophage-derived peroxynitrite diffusion and toxicity to *Trypanosoma cruzi*.. Arch Biochem Biophys.

[32] Holscher C, Kohler G, Muller U, Mossmann H, Schaub GA (1998). Defective nitric oxide effector functions lead to extreme susceptibility of *Trypanosoma cruzi*-infected mice deficient in gamma interferon receptor or inducible nitric oxide synthase.. Infect Immun.

[33] Saeftel M, Fleischer B, Hoerauf A (2001). Stage-dependent role of nitric oxide in control of Trypanosoma cruzi infection.. Infect Immun.

[34] Wrightsman R, Krassner S, Watson J (1982). Genetic control of responses to *Trypanosoma cruzi* in mice: multiple genes influencing parasitemia and survival.. Infect Immun.

[35] Trischmann TM, Bloom BR (1982). Genetics of murine resistance to *Trypanosoma cruzi*.. Infect Immun.

[36] Aliberti JC, Souto JT, Marino AP, Lannes-Vieira J, Teixeira MM (2001). Modulation of chemokine production and inflammatory responses in interferon-gamma- and tumor necrosis factor-R1-deficient mice during *Trypanosoma cruzi* infection.. Am J Pathol.

[37] Kayama H, Koga R, Atarashi K, Okuyama M, Kimura T (2009). NFATc1 mediates Toll-like receptor-independent innate immune responses during *Trypanosoma cruzi* infection.. PLoS Pathog.

[38] Machado AV, Cardoso JE, Claser C, Rodrigues MM, Gazzinelli RT (2006). Long-term protective immunity induced against *Trypanosoma cruzi* infection after vaccination with recombinant adenoviruses encoding amastigote surface protein-2 and trans-sialidase.. Hum Gene Ther.

[39] Tzelepis F, Persechini PM, Rodrigues MM (2007). Modulation of CD4+ T cell-dependent specific cytotoxic CD8+ T cells differentiation and proliferation by the timing of increase in the pathogen load.. PLoS ONE.

[40] Silva GK, Gutierrez FRS, Guedes PMM, Horta CV, Cunha LD (2009). Cutting Edge: Nucleotide-Binding Oligomeriz*ation Domain 1-Dependent Responses Account for Murine Resistance against Trypanosoma cruzi* Infection.. J Immunol.

[41] Monteiro AC, Schmitz V, Morrot A, de Arruda LB, Nagajyothi F (2007). Bradykinin B2 Receptors of dendritic cells, acting as sensors of kinins proteolytically released by *Trypanosoma cruzi*, are critical for the development of protective type-1 responses.. PLoS Pathog.

[42] Gallucci S, Lolkema M, Matzinger P (1999). Natural adjuvants: endogenous activators of dendritic cells.. Nat Med.

[43] Luft T, Pang KC, Thomas E, Hertzog P, Hart DN (1998). Type I IFNs enhance the terminal differentiation of dendritic cells.. J Immunol.

[44] Curtsinger JM, Valenzuela JO, Agarwal P, Lins D, Mescher MF (2005). Type I IFNs provide a third signal to CD8 T cells to stimulate clonal expansion and differentiation.. J Immunol.

[45] Lang PA, Cervantes-Barragan L, Verschoor A, Navarini AA, Recher M (2009). Hematopoietic cell-derived interferon controls viral replication and virus-induced disease.. Blood.

[46] Costa VM, Torres KC, Mendonca RZ, Gresser I, Gollob KJ (2006). Type I IFNs stimulate nitric oxide production and resistance to *Trypanosoma cruzi* infection.. J Immunol.

[47] Egan CE, Sukhumavasi W, Butcher BA, Denkers EY (2009). Functional aspects of Toll-like receptor/MyD88 signalling during protozoan infection: focus on *Toxoplasma gondii*.. Clin Exp Immunol.

[48] Sukhumavasi W, Egan CE, Warren AL, Taylor GA, Fox BA (2008). TLR adaptor MyD88 is essential for pathogen control during oral *Toxoplasma gondii* infection but not adaptive immunity induced by a vaccine strain of the parasite.. J Immunol.

[49] Bolz DD, Sundsbak RS, Ma Y, Akira S, Kirschning CJ (2004). MyD88 plays a unique role in host defense but not arthritis development in Lyme disease.. J Immunol.

[50] von Bernuth H, Picard C, Jin Z, Pankla R, Xiao H (2008). Pyogenic bacterial infections in humans with MyD88 deficiency.. Science.

[51] Way SS, Kollmann TR, Hajjar AM, Wilson CB (2003). Cutting edge: protective cell-mediated immunity to *Listeria monocytogenes* in the absence of myeloid differentiation factor 88.. J Immunol.

[52] Shi S, Nathan C, Schnappinger D, Drenkow J, Fuortes M (2003). MyD88 Primes Macrophages for Full-Scale Activation by Interferon-{gamma} yet Mediates Few Responses to *Mycobacterium tuberculosis*.. J Exp Med.

[53] Tarleton RL, Grusby MJ, Postan M, Glimcher LH (1996). *Trypanosoma cruzi* infection in MHC-deficient mice: further evidence for the role of both class I- and class II-restricted T cells in immune resistance and disease.. Int Immunol.

[54] Kumar S, Tarleton RL (1998). The relative contribution of antibody production and CD8+ T cell function to immune control of *Trypanosoma cruzi*.. Parasite Immunol.

[55] Pasare C, Medzhitov R (2005). Control of B-cell responses by Toll-like receptors.. Nature.

[56] Gavin AL, Hoebe K, Duong B, Ota T, Martin C (2006). Adjuvant-enhanced antibody responses in the absence of toll-like receptor signaling.. Science.

[57] Browne EP, Littman DR (2009). Myd88 is required for an antibody response to retroviral infection.. PLoS Pathog.

[58] Heer AK, Shamshiev A, Donda A, Uematsu S, Akira S (2007). TLR signaling fine-tunes anti-influenza B cell responses without regulating effector T cell responses.. J Immunol.

[59] Guay HM, Andreyeva TA, Garcea RL, Welsh RM, Szomolanyi-Tsuda E (2007). MyD88 is required for the formation of long-term humoral immunity to virus infection.. J Immunol.

[60] Nakanishi Y, Lu B, Gerard C, Iwasaki A (2009). CD8+ T lymphocyte mobilization to virus-infected tissue requires CD4+ T-cell help.. Nature.

[61] Pereira da Silva LH, Nussenzweig V (1953). Sobre uma cepa de *Trypanosoma cruzi* altamente virulenta para o camundongo branco.. Fol Clin Biol.

[62] Green LC, Wagner DA, Glogowski J, Skipper PL, Wishnok JS (1982). Analysis of nitrate, nitrite, and [15N]nitrate in biological fluids.. Anal Biochem.

[63] de Alencar BC, Araujo AF, Penido ML, Gazzinelli RT, Rodrigues MM (2007). Cross-priming of long lived protective CD8+ T cells against *Trypanosoma cruzi* infection: importance of a TLR9 agonist and CD4+ T cells.. Vaccine.

[64] Martin DL, Tarleton RL (2005). Antigen-Specific T Cells Maintain an Effector Memory Phenotype during Persistent *Trypanosoma cruzi* Infection.. J Immunol.

