# Supplementary figures and images for: Impaired Innate Immunity in Tlr4 −/− Mice but Preserved CD8+ T Cell Responses against Trypanosoma cruzi in Tlr4-, Tlr2-, Tlr9- or Myd88-Deficient Mice
Source: PLoS Pathog. 2010 Apr 29;6(4):e1000870. doi: 10.1371/journal.ppat.1000870 (PMC2861687; doi:10.1371/journal.ppat.1000870)

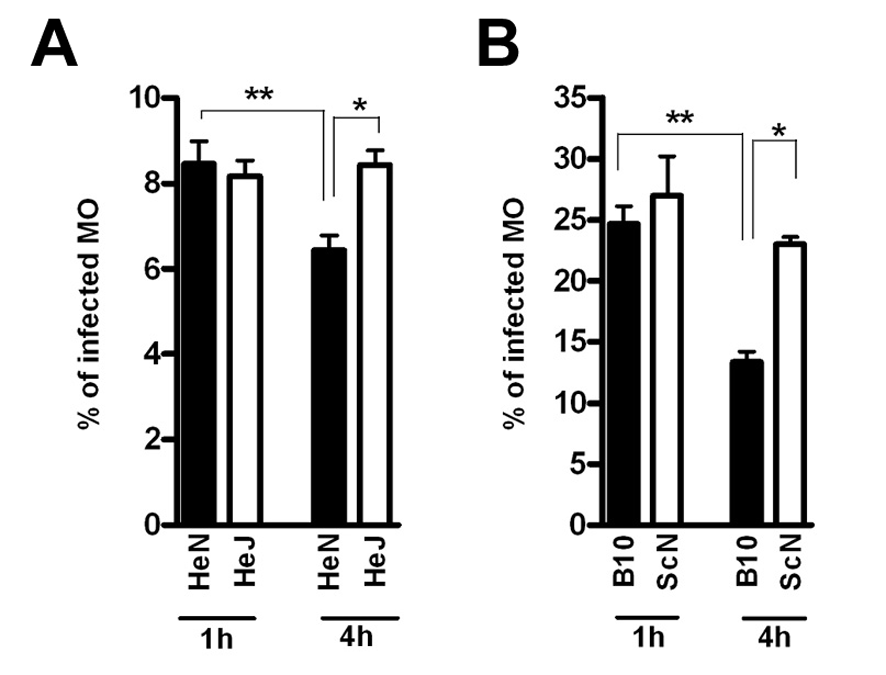

Supplement: Figure S1 — Early trypanosomacidal mechanism is absent in TLR4-deficient macrophages. (A) Resident macrophages from C3H/HePas (wt, black bars) and C3H/HeJ (Tlr4d/d, white bars) mice were infected with blood form trypomastigotes of the Y strain in a 1∶10 (macrophage:trypomastigotes) ratio, for 1 h. After this period, extracellular trypomastigotes were removed by washing and the cells were fixed and stained with Giemsa (1 h). Alternatively, after washing, cultures were prolonged for a total 4-h incubation time, after which cells were fixed and stained with Giemsa (4 h). (B) Resident macrophages from C57BL/10 (wt, black bars) and C57BL/10ScN (Tlr4−/−, white bars) were infected and treated as in (A). The percentage of infected macrophages was counted under a light microscope and each data point is expressed as the mean + SEM of triplicates. Experiments shown are representative of at least two independent experiments. One asterisk (*) indicates that the percentage of infected MO is significantly different (p<0.05) between WT and TLR4-deficient MO after the 4 h period of culture. Two asterisks (**) indicate that the percentage of infected MO is significantly different (p<0.05) between WT MO cultivated by 1 h and 4 h periods of culture. (0.61 MB TIF) [file ppat.1000870.s001.tif]

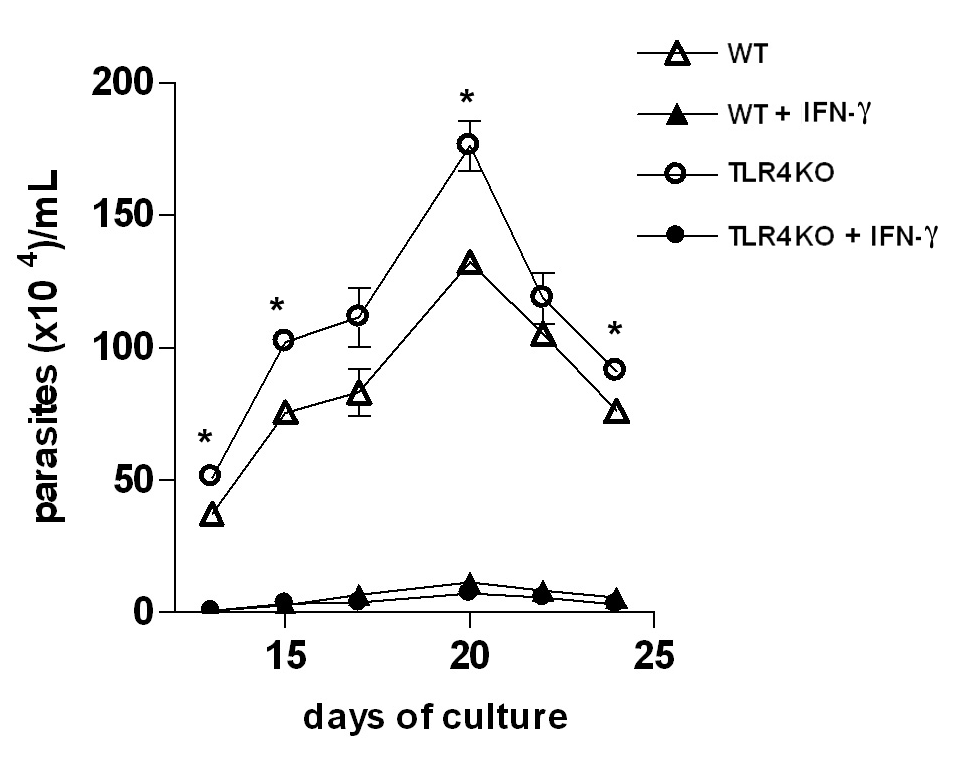

Supplement: Figure S2 — IFN-γturns WT and Tlr4−/− MO equally resistant to infection with T. cruzi. 105 resident peritoneal macrophages from Tlr4−/− mice and from their WT controls (C57BL/6) were cultured in the presence of trypomastigotes of the Y strain in a 1∶10 (macrophage:trypomastigotes) ratio, for 1 h. After removal of extracellular trypomastigotes by extensive washing, cultures were continued for several days and the number of trypomastigotes released into the supernatants was determined daily from day 10 on. In some triplicates, rmIFN-γ (2.0 ng/ml) was added to the cultures from the beginning (black symbols). Asterisks (*) indicate that the number of trypomastigotes released into the supernatants is significantly different (p<0.05) between WT and TLR4-deficient MO cultures. Each data point is expressed as the mean ± SEM of triplicates and experiments shown are representative of at least two independent experiments. (0.76 MB TIF) [file ppat.1000870.s002.tif]
